# First isolation, genetic characterization, and predicted antigenic features of lumpy skin disease virus from clinically affected cattle in Tanzania reveal divergence between field and vaccine strains

**DOI:** 10.5455/javar.2026.m1064

**Published:** 2026-09-11

**Authors:** Paskalina Badi Niima, Alexanda Danford Mzula, Philemon Nyangi Wambura, Gabriel Mkilema Shirima

**Affiliations:** 1 College of Science and Technical Education, Mbeya University of Science and Technology, Mbeya, Tanzania; 2 School of Life Sciences and Bioengineering, The Nelson Mandela African Institution of Science and Technology, Arusha, Tanzania; 3 College of Veterinary Medicine and Biomedical Sciences, Sokoine University of Agriculture, Morogoro, Tanzania

**Keywords:** cattle, phylogenetic analysis, Tanzania, EEV126 gene, antigenic variation, Lumpy skin disease virus

## Abstract

Objectives: This study aimed to isolate the lumpy skin disease virus (LSDV) from cattle in Tanzania and determine predicted antigenic features between the vaccine strain and circulating field strains. Materials and Methods: Skin nodules were aseptically collected from clinically affected cattle. The LSDV was isolated from embryonated chicken eggs (ECE) and the Vero cells. The extracellular enveloped virus (EEV126) gene was targeted for molecular detection using polymerase chain reaction (PCR). For phylogenetic and in silico analysis, PCR-positive products from the field samples and isolated virus were partially sequenced, targeting the EEV126 gene. Results: The virus induced cytopathic effects from the first passage in Vero cells and typical lesions on the chorioallantoic membrane (thickening, hemorrhage, edema, and pock lesions). Phylogenetic analysis of nucleotide and deduced amino acid sequences revealed that Tanzanian field strains clustered closely with the reference sequences from India, Egypt, Bangladesh, and Bhutan, suggesting shared genetic and antigenic characteristics. Additionally, the field strains were genetically distinguished from reference vaccines as well as the vaccine commercially used in Tanzania, indicating genetic and antigenic variation. The identified conserved epitope and its surface exposure on the 3D structure may indicate a potential cross-reactive immune recognition across LSDV strains. Conclusions: This study successfully isolated LSDV from Tanzanian cattle and revealed antigenic variation between the circulating field strains and vaccine strain, particularly within predicted antigenic regions of the EEV126 gene. Also, the identified conserved surface-exposed epitope may serve as a potential target for cross-reactive immune recognition.

## 1. Introduction

Lumpy skin disease virus (LSDV) is an enveloped, linear double-stranded DNA virus of the genus Capripoxvirus in the family *Poxviridae* [1]. The LSDV has a genome of about 151,000 base pairs (bp), consisting of a central coding region that contains 156 putative genes [1]. The LSDV causes LSD in cattle, a highly contagious viral infection that leads to substantial economic losses for the livestock industry through reduced milk production, trade restrictions, skin damage, weight loss, and infertility in bulls [2, 3]. The disease is primarily transmitted mechanically by blood-feeding vectors, such as the stable fly (*Stomoxys calcitrans*) [4, 5]. In addition, experimental studies have confirmed that other vectors, such as male ticks (*Rhipicephalus appendiculatus*) and mosquitoes (*Aedes aegypti*), are capable of transmitting the disease [6]. The disease is characterized by high temperature, skin nodules, nasal and oral discharge, salivation, loss of appetite, depression, reluctance to move, enlargement of lymph nodes, and swelling of limbs [7]. The LSD was first identified in Zambia in 1929; thereafter, the disease has spread across Africa, with recent occurrences documented in the Middle East, Asia, and Europe [8].

The effective management of the disease depends on the presence of seroprevalence and molecular epidemiological information, which guides control strategies [9]. There are various strategies used to control the spread of LSD, such as early findings of outbreaks, complete or partial eradication, restrictions on animal movement, quarantine measures, and mass vaccination campaigns. Vaccination with the live attenuated Neethling vaccine has been the most widely used and effective method for controlling LSDV in endemic regions [10, 11]. In Tanzania, vaccination against lumpy skin disease is predominantly carried out using live attenuated vaccines, which are locally produced and also imported from neighboring countries, particularly Kenya. Lumpy skin disease remains a persistent threat to cattle production in Tanzania, resulting in significant economic losses at both household and national levels.

In Tanzania, the first LSD occurrence was reported between 1981 and 1986, with a mortality rate of around 20% in affected cattle [12, 13]. Research on LSDV in Tanzania is limited in scope, with most studies focusing on diagnostics, seroprevalence, disease patterns, and molecular detection [14–19]. The previous studies seem to be focused on the validation and performance via routine diagnostic methods for LSDV, such as real-time PCR, LAMP, and mobile PCR [18, 19]. Additionally, studies conducted in the eastern and southern highland zones of Tanzania reported LSDV seroprevalence ranging from 13% to 18% at the animal level and 22.7% to 44.6% at the herd level [15, 16]. As well, a spatial and temporal analysis of LSD outbreaks in Tanzania has been conducted, focusing on the general trends of LSD outbreaks between 2018 and 2023 [14]. Moreover, the molecular study in Tanzania documented the circulation of LSDV strains in Tanga, Pwani, and Rukwa [17].

The former study has documented several structural proteins of LSDV that are antigenic, including *LSDV60*, *LSDV117*, *LSDV126* (also referred to as *EEV126*), and *LSDV141*, that play a key role in viral structure and are important targets for host immune responses [20]. The present study selected *EEV126* as it contains a 9-amino acid insertion that serves as a molecular marker for differentiating vaccine strains from field isolates, making it valuable for evaluating host immune responses and monitoring circulating virus variants [21]. Isolation of LSDV in embryonated chicken eggs and continuous cell lines was documented by several former studies, highlighting the understanding of the characteristics of the virus, supporting further virological studies, and being used as vaccine candidates for developing an effective control of lumpy skin disease [22–25].

Despite existing research on LSDV diagnostics and epidemiology in Tanzania, there have been no documented reports regarding LSDV isolation and its antigenic features. In particular, there is a lack of information on the antigenic profiles of the field LSDV strains compared to the vaccine strains, as defined by their epitopes, which is crucial for understanding immune recognition and vaccine effectiveness. Thus, this study aimed to isolate LSDV and determine its antigenic features to gain an understanding of immune recognition and vaccine efficacy.

## 2. Materials and Methods

### 2.1. Ethical approval

The ethical approval for this study was granted by the Kibongoto-Nelson Mandela-CEDHA Health Research Ethics Committee (KNCHREC) (Approval No. KNCHREC00009/09/2023). All official communication regarding the study was conducted through livestock extension officers at the time of sample collection.

### 2.2. Study area and sample collection

Samples were purposively collected from cattle suspected of LSD during outbreak investigations conducted in nine districts of Tanzania: Meru, Arusha District, Babati, Bahi, Dodoma City Council, Misungwi, Sengerema, Musoma, and Kasulu. This study formed part of a broader molecular epidemiological investigation of LSDV associated with these outbreaks. A total of 33 blood samples and 33 skin biopsy samples were purposively collected from clinically suspected cattle. Molecular screening confirmed 20 skin biopsy samples as LSDV-positive by PCR. Subsequent molecular characterization of the *GPCR* gene revealed amino acid sequence variation among the twenty positive samples. Six isolates exhibited amino acid substitutions, originating from the districts of Bahi (*n* = 2), Musoma (*n* = 2), Sengerema (*n* = 1), and Meru (*n* = 1). The remaining 14 isolates showed no amino acid substitutions. These were distributed across the districts of Meru (*n* = 7), Bahi (*n* = 2), Musoma (*n* = 1), Babati (*n* = 1), Sengerema (*n* = 1), Misungwi (*n* = 1), and Arusha DC (*n* = 1). Based on this genetic grouping, representative skin biopsy samples (*n* = 3) were purposively selected for virus isolation and in silico analysis of antigenic features. Specifically, one representative isolate showing amino acid substitution was selected from Bahi district, while two representative isolates with no substitutions were selected from Meru and Babati districts. Selection was based on representativeness of the observed *GPCR* amino acid sequence variation, ensuring inclusion of both amino acid substitution and non-substitution groups across districts.

### 2.3. LSDV isolation and propagation

#### 2.3.1. Skin biopsy sample processing and LSDV inoculum preparation

The LSDV isolation was performed in the chorioallantoic membrane (CAM) of 10-day-old embryonated chicken eggs and in Vero cell culture [24, 26]. Skin biopsy samples were thawed at room temperature, washed three times in phosphate-buffered saline (PBS), and homogenized using a sterile mortar and pestle [25]. The homogenates were suspended in PBS containing antibiotics to prepare 20% (2 gm of tissue in 8 ml of PBS) tissue suspensions. The suspensions were centrifuged at 3000 rpm for 15 min, and the supernatant was filtered through a 0.45 µm sterile nylon membrane for virus inoculation.

#### 2.3.2. LSDV isolation in embryonated chicken eggs (CAM)

Fertilized chicken eggs were obtained from a certified commercial hatchery and incubated under standard laboratory conditions for 10 days to obtain 10-dayold embryonated eggs (ECE) prior to virus isolation. The ECEs were divided into experimental, positive control, and negative control groups. The experimental group was inoculated with filtered LSDV field sample suspensions (*n* = 3 from Bahi, Babati, and Meru districts); the positive control, with a live attenuated Neethling LSDV vaccine strain; and the negative control, with sterile PBS containing antibiotics. Five 10-day-old ECE were used per group. A volume of 100 µl of each inoculum was inoculated onto the CAM of 10-day-old ECE under sterile conditions, as previously described [26, 27]. The inoculated eggs were incubated at 37 °C and examined daily using an egg candler for 5 days to assess embryo viability. At 5 days post-inoculation, eggs were chilled at 2-8 °C overnight. The CAMs were harvested, homogenized using a sterile mortar and pestle, and centrifuged at 3000 rpm for 15 min. The supernatant was collected, aliquoted, and stored at -80 °C.

Virus isolation was performed in three independent experimental replicates for each isolate. In each replicate, initial inoculation and two serial passages were carried out, where supernatant from the previous passage was used to inoculate fresh ECE following the same procedure. Positive and negative controls were maintained and passaged in parallel with field samples across all experimental replicates and subsequent passages under identical conditions.

#### 2.3.3. LSDV isolation in Vero cells

Vero cells used in the present study were provided in kind by Ifakara Health Institute, Department of Biomedical Research and Clinical Trials (Bagamoyo Branch). Cells were utilized for LSDV isolation as they support viral propagation, as described in previous studies [22, 24, 28]. Vero cells were cultured in 25 cm² tissue culture flasks to obtain a confluent monolayer. Briefly, cells were seeded in Minimum Essential Medium (MEM) supplemented with 10% fetal bovine serum (FBS) and 1% penicillin-streptomycin and incubated at 37 °C in a humidified 5% CO₂ atmosphere for 72 h.

For inoculation, confluent monolayers were washed three times with sterile PBS. A total of 500 µl of LSDV inoculum derived from field samples (*n *= 3 from Bahi, Babati, and Meru districts) was added and allowed to adsorb for 1 h at 37 °C in 5% CO₂. After adsorption, 7 ml of maintenance medium (MEM supplemented with 2% FBS and 1% penicillin-streptomycin) was added, and cells were further incubated under the same conditions. A live-attenuated Neethling vaccine strain was used as a positive control, while uninfected cells served as negative controls.

Cells were examined daily for 7 days under an inverted microscope to detect virus-induced cytopathic effects (CPE). Observation of CPE in infected cultures was considered evidence of LSDV infection. When CPE was observed, the virus was harvested by three freeze-thaw cycles to lyse cells and release viral particles, and the supernatant was stored at -80 °C for further analysis.

Virus isolation in Vero cells was performed in three independent experimental replicates, with initial inoculation and two serial passages (P0-P2) conducted over 7 days. For each passage, 500 µl of harvested supernatant was used to infect fresh cell monolayers following the same procedure. Positive and negative controls were included and passaged in parallel with field samples under identical conditions throughout the experiment.

#### 2.3.4. DNA extraction and conventional PCR

DNA was extracted using the Tissue and Blood DNeasy Kit (Qiagen, Germany), following the manufacturer's instructions. A total of 58 samples were used for DNA extraction, comprising 3 field skin biopsy tissue suspensions and 1 commercially available LSDV Neethling vaccine strain prior to inoculation. Post-inoculation samples included 27 CAM isolates and 27 infected Vero cell culture isolates generated across three independent experimental replicates following initial inoculation and subsequent passages. Positive and negative controls were included in all experimental runs for assay validation. A commercially available live attenuated LSDV Neethling vaccine (*Lumpy Skin Disease Vaccine, Live, Neethling strain*, Batch/Lot No. 7007) served as a positive control, and nuclease-free water was used as a negative control. The DNA was eluted in 100 μl of elution buffer, and its concentration was measured using a Nanodrop, while its quality was assessed by gel electrophoresis. The DNA samples were then stored at -20 °C.

PCR amplification targeting the external envelope glycoprotein gene (*EEV126*) was performed using OneTaq ® Quick-Load ® 2X Master Mix (New England Biolabs, USA) according to the manufacturer's instructions. The specific primers targeting the *EEV126* gene (forward: 5ʹ-ATG GGA ATA GTA TCT GTT GTA TAC G-3ʹ; reverse: 5ʹ-CGA ACC CCT ATT TAC TTG AGA A-3ʹ) were used to generate a 958 bp amplicon, as previously described [29]. Optimization of annealing conditions was performed using gradient PCR with 1 µl, 3 µl, and 5 µl DNA templates, each in duplicate. The optimal condition was 50 °C with 3 µl of template based on consistent amplification.

PCR reactions were prepared in a final volume of 25 µl containing 12.5 µl master mix, 0.5 µl of each primer, 3 µl DNA template, and 8.5 µl nuclease-free water. A total of 58 DNA samples were subjected to PCR amplification. Amplification was carried out in a Bio-Rad thermal cycler with an initial denaturation at 94 °C for 3 min, followed by 34 cycles consisting of denaturation at 94 °C for 30 sec, annealing at 50 °C for 45 sec, and extension at 68 °C for 1 min. A final extension was performed at 68 °C for 5 min, and the reaction was held at 4 °C.

All reactions were performed in duplicate with appropriate positive and negative controls. PCR products were separated on a 1.5% agarose gel prepared in Tris-borate-EDTA (TBE) buffer, using a 100 bp DNA ladder as a size marker. Gels were stained with SafeView DNA stain and visualized under a UV transilluminator to confirm LSDV based on the expected band size of 958 bp.

### 2.4. Nucleotide sequencing

Out of the 58 PCR-amplified samples, a total of 10 PCR amplicons (1 commercially available LSDV Neethling vaccine strain, 3 field isolates from Bahi, Meru, and Babati districts, 3 infected CAM isolates, and 3 infected cell culture isolates) were selected and sent to Macrogen (Amsterdam, Netherlands) for purification and sequencing. The CAM and cell culture isolates, obtained from the final passage, were selected from a single representative replicate, as biological replicates demonstrated consistent and identical PCR amplification results. Amplicons were purified using Macrogen's EZ-Seq purification system and sequenced using the BigDye Terminator Cycle Sequencing Kit (Applied Biosystems, USA) on an ABI 3730xl Genetic Analyzer. The same forward and reverse primers used for *EEV126* gene amplification were employed for sequencing. Bidirectional sequencing was performed to ensure complete coverage of the 958 bp of the *EEV126* region, and forward and reverse reads were assembled to generate high-quality consensus sequences.

### 2.5. Sequence analysis

Sequence analysis was performed to evaluate genetic variation, infer phylogenetic relationships with reference sequences, and examine potential antigenic differences between field and live attenuated Neethling vaccine strains.

Raw DNA sequence data were obtained from Macrogen and processed in Geneious Prime version 2025.2.2, where low-quality regions were trimmed, sequences were edited, and de novo assembly was used to generate consensus sequences. These were manually inspected against chromatograms to ensure sequence accuracy. Consensus sequences were submitted to Basic Local Alignment Search Tool (BLAST) analysis against the National Center for Biotechnology Information (NCBI) database to determine sequence identity with the reference sequence database available in GenBank. Following BLAST analysis, LSDV reference sequences were selected according to the nucleotide similarity and the isolates' origin. Multiple sequence alignment was performed using ClustalW, which is implemented in MEGA software version 12.

Following alignment, it was observed that 9 sequences (3 field isolates and 6 CAM and Vero cell culture isolates) were identical at both nucleotide and amino acid levels. Therefore, to avoid redundancy in phylogenetic analysis, all three field isolates were retained, while one representative sequence each from CAM isolates and Vero cell culture isolates was selected, together with the vaccine strain, for phylogenetic tree construction.

Sequence identity and genetic distances were calculated using Geneious Prime and MEGA software, respectively.

Representative LSDV vaccine reference strains and other LSDV reference sequences were retrieved from the GenBank database. Nucleotide sequences were translated into amino acid sequences using the ExPASy Translate tool. Multiple sequence alignment in MEGA software was used to compare Tanzanian field isolates, CAM-derived isolates, Vero cell isolates, the commercially available vaccine strain used in Tanzania, reference vaccine strains, and other LSDV reference sequences. Phylogenetic analysis was conducted in MEGA software version 12 using the maximum likelihood method. The Jones-Taylor-Thornton (JTT) substitution model was applied for amino acid sequences, and the Tamura 3-parameter model was used for nucleotide sequences, as described by Kumar et al. [30]. Tree robustness was assessed using 1,000 bootstrap replicates.

### 2.6. Structural and immunoinformatic analysis of the EEV126 gene

The epitope mapping was performed to identify and compare antigenic regions recognized by the immune system in LSDV field isolates and a commercially available live attenuated Neethling vaccine strain. Only one representative field isolate was selected for immunoinformatic analyses because all field isolates showed identical nucleotide and amino acid sequences with no variation among themselves. Therefore, a single representative field sequence was used alongside the vaccine strain for downstream structural and epitope prediction analyses.

The three-dimensional structure of the EEV126 protein was predicted using AlphaFold2 via the ColabFold v1.5.5 pipeline, which automatically generates multiple sequence alignments using MMseqs2. Default parameters were used for structure prediction. Model confidence was assessed using predicted Local Distance Difference Test (pLDDT) scores, encoded in the B-factor column of the Protein Data Bank (PDB) files. The top-ranked structural model (ranked_0.pdb), showing the highest overall confidence, was selected for downstream analyses.

B-cell epitope prediction was performed using the ElliPro tool from the Immune Epitope Database (IEDB), which identifies both linear and conformational epitopes. Predicted B-cell epitope residues were mapped onto the AlphaFold2-generated protein structure using PyMOL ™ Molecular Graphics System (version 2.3.0) in an Ubuntu Linux environment. Custom PyMOL scripts were used to visualize the structure, color residues according to pLDDT scores, and highlight predicted epitope regions. Only residues with high confidence (pLDDT ≥ 80) and ElliPro epitope scores ≥ 0.8 were included in the final epitope mapping analysis.

## 3. Results

### 3.1. LSDV isolation in CAM of embryonated chicken eggs

In the initial inoculation and first passage, embryos from all inoculated eggs remained viable for up to 5 days PI (Table 1). From the second passage, two embryos from one sample died on day 4 PI. The CAMs from these eggs were chilled and harvested for further analysis. For all scenarios, the CAM from the tested samples was harvested, and the inoculated samples were found to induce lesions on CAMs from the initial inoculation to the second passage. The collected CAMs revealed hemorrhage and membrane thickening in the initial inoculation, whereas the pock lesions (white spots), edema, hemorrhage, and membrane thickening were observed in the 1st and 2nd passages across all three experimental replicates (Figure 1). Since all replicates showed consistent results, only one representative replicate from the final passage was selected for sequencing. LSDV infection was confirmed by conventional gel-based PCR in all CAM isolates (*n* = 27 in total, derived from three experimental replicates) across the initial inoculation and subsequent serial passages. In negative control eggs, the embryos were found to be alive in all passages, CAM showed no characteristic pock lesion, and PCR results were negative, whereas positive control eggs showed typical CAM lesions and positive PCR results (Figures 1, 2).

### 3.2. LSDV isolation in Vero cells

The skin biopsy samples (*n* = 3) were inoculated onto Vero cell lines for isolation and propagation of LSDV. Cell cultures were monitored under an inverted microscope and compared with a control flask containing a monolayer of uninfected Vero cells. Vero cells were examined daily for 7 days after inoculation. In the flask inoculated with LSDV filtrate, the cytopathic effects started to appear on the 4th day during the initial inoculation and on the 2nd day during the first and second passages for all experimental replicates, indicating an earlier onset of cytopathic effects in subsequent passages. The observed cytopathic effects (CPE) were evident as cell rounding, cell aggregation, and cell detachment from the confluent monolayer, indicating viability of the virus (Figure 3). Conversely, the cells in the control flask showed normal morphology for Vero cells and remained adherent to the surface of the culture flask. LSDV infection on Vero cells was confirmed by conventional PCR in a total of 27 cell culture isolates derived from three experimental replicates across the initial inoculation and subsequent passages. PCR results were positive in flasks showing cytopathic effects (CPE), whereas the uninfected flask showed PCR negative (Figure 2). Since all experimental replicates showed consistent results, only one representative replicate from the final passage was selected for sequencing.

### 3.3. Sequencing and phylogenetic analysis of the EEV126 gene

The field isolates and re-isolated viruses in CAM and Vero cells were sequenced and successfully uploaded to GenBank (accession numbers PX496892 to PX496896). Multiple sequence alignment between the commercially available vaccine and Tanzanian isolates obtained from this study revealed that the percentage identity ranges from 92.21% to 100% (Table 2), indicating that most amino acid residues in the *EEV126 *gene have been conserved. Despite a high amino acid identity of 92.21-100% between vaccine strains and the Tanzanian isolates, a small proportion (7.79%) of amino acid residues differed between them, which could potentially affect antigenicity and may compromise vaccine function. In comparison with LSDV Neethling vaccine virus strains, the Tanzanian field isolates showed amino acid substitutions E→K at positions 60 and 85 and G→S at position 100, highlighting sequence variation between them and possibly suggesting ongoing virus evolution (Figure 4).

Additionally, the sequence alignment unveiled the insertions of a 9-amino-acid (9-aa) sequence (SVYDLPPND) within the tandem repeat region of the *EEV126 *gene (positioned 133-141) (Figure 4). The Tanzanian LSDV strains possess two copies of 9-aa (corresponding to 27 nucleotides), which was also present in the LSDV KSGP-0240-derived vaccines, whereas the commercially available LSDV vaccine contained only a single copy (Figure 4).

These variations, resulting from amino acid substitutions and the 9-amino acid insertion in the field strains, may change antigenic properties. The insertion of 9-amino acids in the tandem repeat region resulted in differences in protein length, with the Tanzanian strain having 181 amino acids and the commercially available LSDV vaccine containing 172 amino acids.

The phylogenetic analysis based on nucleotide sequences revealed that LSDV clustered into two distinct groups, including vaccine strains and field virulent strains (Figure 5). The vaccine strains formed a distinct clade, whereas the Tanzanian field strains grouped into a separate clade. This indicates close genetic relatedness among circulating field isolates and genetic divergence from the vaccine strains.

The commercially available vaccines (positive control) used in Tanzania clustered with reference LSDV Neethling-derived vaccine strains and formed a separate clade from the field isolates, indicating that Tanzanian field strains were genetically different from the vaccines used for animal vaccination in the country. Conversely, the Tanzanian field strains clustered closely with the reference sequences from India, Egypt, Bangladesh, and Bhutan, suggesting close genetic characteristics. Moreover, the Tanzanian isolates and the Kenyan vaccine strain (Kenyavac KSGP 0240) grouped within the same clade, indicating genetic relatedness (Figure 5).

The phylogenetic analysis based on amino acid sequences revealed that all LSDV sequences, including Tanzanian LSDV strains, commercially available vaccine (positive control) strains, reference LSDV Neethling vaccines, and other reference strains, belong to a single clade (Figure 6). The grouping of sequences within a single clade highlights their overall similarity, reflecting that EEV126 amino acid sequences were highly conserved across all strains.

### 3.4. Structural and immunoinformatics of the EEV126 protein of LSDV

Structural and immunoinformatic analyses of the *EEV126* gene were performed to provide an understanding of how sequence variations among LSDV strains may affect protein conformation, immune recognition, and the potential effectiveness of currently available vaccines.

### 3.5. Prediction of B-cell epitope

ElliPro analysis identified three main surface-exposed regions, potentially antigenic regions indicated in yellow, with protrusion index (PI) scores above the threshold value of 0.5 in the *EEV126 *gene of the LSDV vaccine strain (Figure 7). The regions located at amino acid residues 34-46 and 142-169 have scores above 0.6, indicating that these surface-exposed regions are likely to contain B-cell epitopes. The broader yellow peak, an extended surface-exposed region spanning amino acid residues 52-138, with scores above 0.5 in the *EEV126 *gene of the LSDV, may represent multiple overlapping predicted B-cell epitopes.

Moreover, a total of four surface-exposed regions indicated in yellow, with scores above the threshold value of 0.5, were revealed in the *EEV126 *gene of the LSDV field strain as predicted by the ElliPro analysis tool (Figure 8). The regions with high scores above 0.6 in field strains were located at amino acid residues 34-46 and 165-178, suggesting B-cell epitopes. In addition, another surface-exposed region with a score above the threshold of 0.5 in the field strain spanned amino acid residues 151-163 and may represent another predicted B-cell epitope that could be recognized by the immune system. Also, the extended surface-exposed region located at amino acid residues 52-147 with the score above 0.5 was identified in the EEV126 protein of the LSDV field strain, representing many overlapping epitopes with the 9-amino acid insertion (SVYDLPPND) (Figure 8).

### 3.6. Epitope sequence conservation

Among the predicted B-cell epitopes, only epitope 1 from vaccine and field strains showed complete conservation of 100% between the two (Figure 9). The high level of sequence conservation (100%) suggests that epitope 1 has fully conserved sequences, with a perfect match between the vaccine-derived amino acid sequences and field strains. The observed 100% sequence conservation of epitope 1 suggests that it was less likely to be affected by strain variation and may therefore serve as a potential candidate for inducing cross-reactive immune response. Epitopes 2 and 3 showed sequence conservation ranging from 0 to 14.29% between field and vaccine strains, indicating that these epitopes are highly variable across strains (Figure 9). This implies that epitopes 2 and 3 are unlikely to induce a cross-reactive immune response and may be strain-specific.

### 3.7. 3D protein structure prediction

The 3D protein structure of the LSDV vaccine and the field strain were predicted using AlphaFold2. AlphaFold2 successfully generated high-quality 3D structural models for both LSDV field strains and vaccine strains. Both protein structures exhibited a higher proportion of residues with pLDDT ≥ 80, indicating overall higher structural confidence.

The mapping of predicted epitopes onto AlphaFold2-derived 3D protein structures for both LSDV vaccine and field strains revealed that all the predicted B-cell epitopes from both LSDV strains were located on the surface-exposed regions of the protein structure (Figures 10A, B). Again, the mapping of epitope 1 (residues 34-46) with 100% sequence conservation revealed a surface-exposed region, suggesting its potential accessibility to immune recognition (Figure 10C).

Moreover, the inserted amino acids (SVYDLPPND at 133-141) in LSDV field strains were mapped onto the overlay structure of the protein and were found to be in a surface-exposed region (Figure 10D). The surface exposure of the inserted 9-aa in LSDV field strains may contribute to antigenic variation in LSDV strains and could potentially facilitate immune escape.

## 4. Discussion

LSDV is a highly contagious cattle pathogen responsible for significant economic losses and is endemic in multiple African and Middle Eastern countries [31, 32]. Given the endemic nature of LSD, this study aimed to isolate LSDV and determine its predicted antigenic features to support ongoing surveillance and vaccination control efforts. Following the isolation of LSDV on the CAM, the observed lesions demonstrate that the virus was viable and infectious. These findings are consistent with previous studies reporting successful LSDV isolation in embryonated chicken eggs [24–26, 33]. Similarly, in Vero cell culture, the appearance of CPE during the initial inoculation is consistent with the previous study᾽s reported appearance of CPE on day five of the first passage [34], indicating successful primary virus isolation in susceptible cells. The development of CPE indicates successful infection and propagation of LSDV in cell culture. Overall, the successful isolation of LSDV using both CAM and Vero cell systems demonstrates the applicability of these methods for viral propagation. The establishment of this protocol provides a useful framework for LSDV isolation and propagation in the study setting.

The variations, resulting from amino acid substitutions and the 9 amino acid insertion in the field strains, may change antigenic properties and could potentially affect immune recognition, as also reported by a former study [21]. A similar 27 bp (9 amino acid) insertion, absent in Neethling vaccine strains and other Capripoxviruses, together with the presence of the SVYDLPPND motif in the *EEV126* gene, has been reported in virulent strains of LSDV found in the field and associated with cattle infection [21]. Additionally, this 9-amino-acid insertion has been reported as a molecular marker distinguishing field isolates from Neethlingderived vaccine strains [35, 36]. In phylogenetic analysis based on nucleotides, the reference vaccine strains and the vaccine used in Tanzania clustered separately from Tanzanian field isolates, suggesting genetic differences among them and supporting the use of the *EEV126 *gene as a molecular marker for differentiating vaccine and field strains. This finding is consistent with previous reports that identified clear genetic separation between vaccine and field isolates [17, 21, 35, 36]. The distinct clustering of Tanzanian isolates from LSDV Neethling-derived vaccines as well as commercially available vaccines suggests that the LSDV outbreaks may not be due to vaccine-derived strains [37].

However, the Tanzanian field strains clustered closely with LSDV isolates from India, Egypt, Bangladesh, and Bhutan, indicating that genetically similar LSDV strains are likely to occur across borders, as also described by a previous study [36]. This clustering suggests a potential risk of continental spread, likely influenced by the movement of animals and animal by-products [36]. The overall clustering of LSDV amino acid sequences from Tanzanian isolates, reference strains, and vaccine strains into a single clade demonstrates the high conservation of the EEV126 amino acid sequences, consistent with a previous study that reported little genetic variation among LSDV isolates [38].

Surface-exposed regions with a threshold above 0.5 were identified in both field and vaccine strains, indicating that these regions are more likely to interact with host antibodies and potentially contribute to immune recognition. A predicted B-cell epitope spanning residues 34-46 was found to be 100% conserved across all analyzed strains and located on the protein surface in the 3D structural model, suggesting accessibility to antibodies and potential as a cross-reactive antigenic target. These findings are consistent with previous reports documenting that surface-exposed and conserved epitopes may play important roles in immune recognition and vaccine design [39–42].

However, experimental validation is required to confirm the immunological relevance and protective potential of this predicted epitope. Importantly, experimental studies on lumpy skin disease virus (LSDV) have demonstrated that protective immune responses involve both humoral and cell-mediated mechanisms, including IFN-γ production, CD8+ T cell activation, and regulatory microRNAs that modulate antiviral immunity [43]. These findings from Kumar et al. [43] indicate that evaluation of predicted epitopes should extend beyond sequence conservation and structural exposure to include functional immunological assays. The presence of overlapping predicted B-cell epitopes within extended surface-exposed regions further suggests a potential immunodominant antigenic site. Additionally, the surface exposure of the nine-amino-acid insertion in field strains may contribute to antigenic variation and influence immune recognition; however, its immunological significance requires further experimental validation. Evidence from other viral studies, such as influenza, suggests that partial immune protection may still occur despite antigenic differences [44].

Taken together, these findings suggest that the conserved B-cell epitope may support immune recognition across analyzed LSDV strains, indicating potential antigenic relevance of the vaccine to circulating viruses. However, sequence variation within multiple overlapping epitope regions suggests differences in antigenic similarity, which may influence vaccine performance. Overall, the results indicate possible partial cross-reactive immune recognition between field and vaccine strains; however, definitive conclusions regarding vaccine efficacy require experimental validation.

## 5. Conclusions

The successful isolation of the lumpy skin disease virus (LSDV) confirms the presence of viable viral strains in Tanzania and provides an important foundation for future vaccine candidate development in the region. Phylogenetic analysis showed that Tanzanian field strains cluster closely with isolates from India, Egypt, Bangladesh, and Bhutan, suggesting circulation of genetically related viruses across geographical boundaries. Importantly, this study identifies a conserved B-cell epitope within the EEV126 protein shared between vaccine and circulating strains, suggesting a potential immunological basis for cross-reactive immune responses and possible partial protection across strains. This finding highlights a potential target for broadly protective LSDV vaccine design. Overall, these results provide new insight into conserved antigenic regions that may be exploited in future vaccine development strategies for LSDV. The study is limited by the lack of experimental validation of predicted antigenic features; therefore, the findings should be interpreted as preliminary and require further immunological confirmation. In addition, continuous molecular surveillance of variable surface regions is recommended to monitor potential antigenic changes, while experimental validation is warranted to confirm the immunological relevance of these computational predictions.

## Figures and Tables

**Figure 1:**
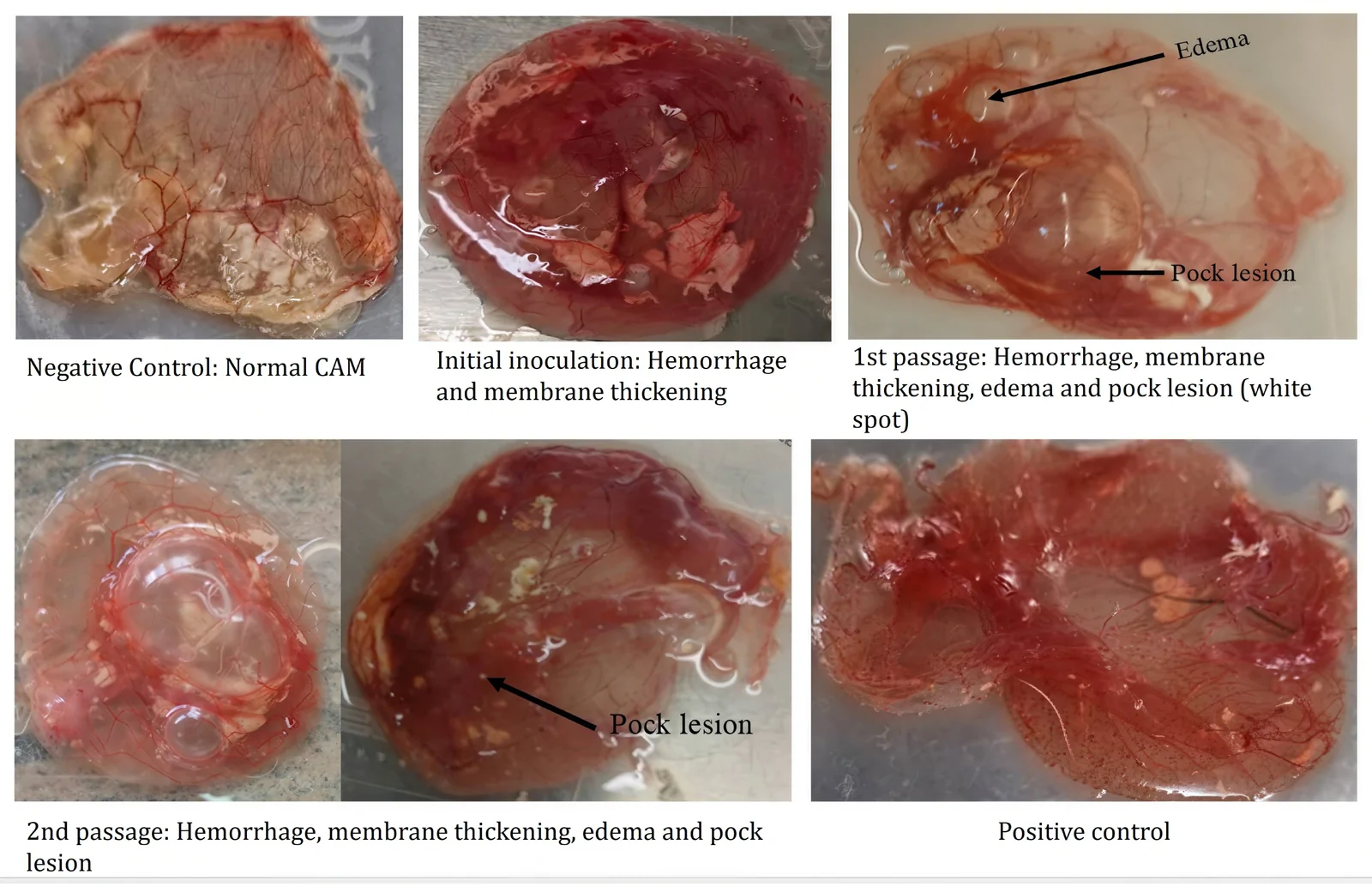
Gross pathological changes observed on the CAM of ECE following inoculation with LSDV. The negative control shows normal CAM morphology without lesions. (Initial inoculation) shows CAM exhibited hemorrhage and membrane thickening. Passages (first and second) show progressive pathological changes, including hemorrhage, membrane thickening, edema, and characteristic pock lesions (white spots), indicating successful viral propagation. The positive control (live attenuated Neethling vaccine strain) showed comparable lesion development. Arrows indicate key pathological features such as edema and pock lesions.

**Figure 2:**
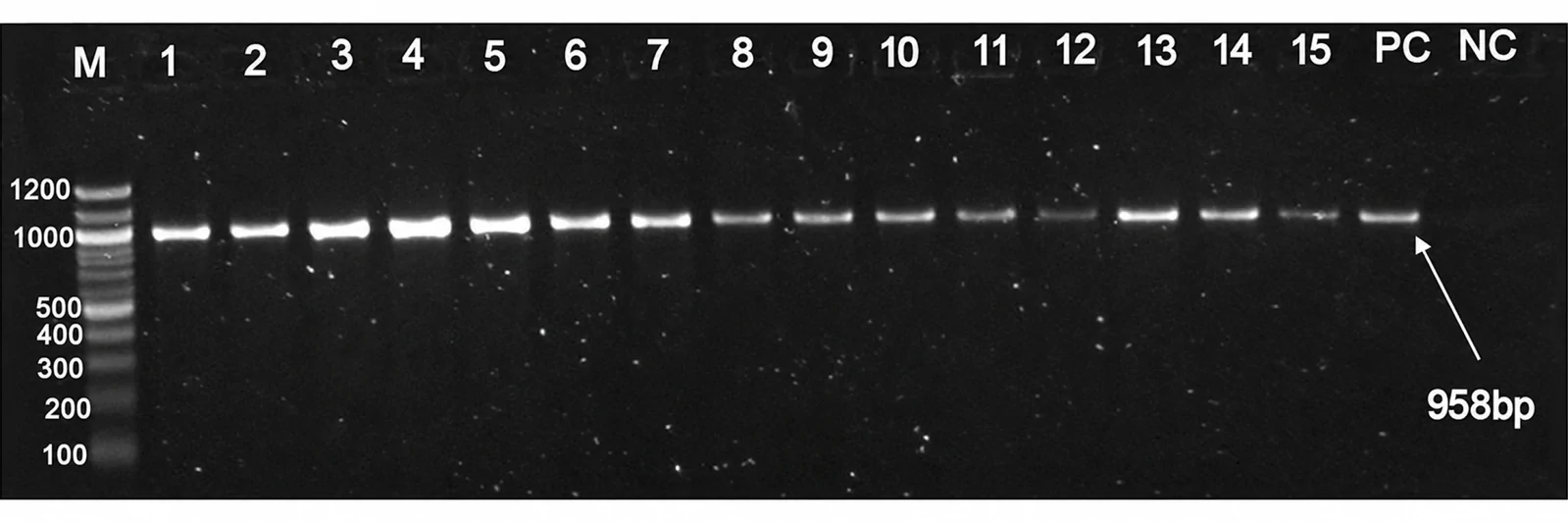
Representative agarose gel electrophoresis showing amplification of the *EEV126* gene of LSDV from field samples and CAM and Vero cell culture isolates at different stages of propagation, along with positive and negative controls.Note: Lane M (100-bp DNA ladder); Lanes 1–3 (tested samples from field cases); Lanes 4–6 (isolates from Vero cell, 1^st^ passage); Lanes 7–9 (isolates from CAM, 1^st^ passage); Lanes 10–12 (CAM isolate, initial inoculation); Lanes 13–15 (Vero cell isolates, initial inoculation); Lane PC (positive control vaccine available in the market (PC/VAC)); and Lane NC (negative control (RNase-free water)). The expected band size is 958 bp.

**Figure 3:**
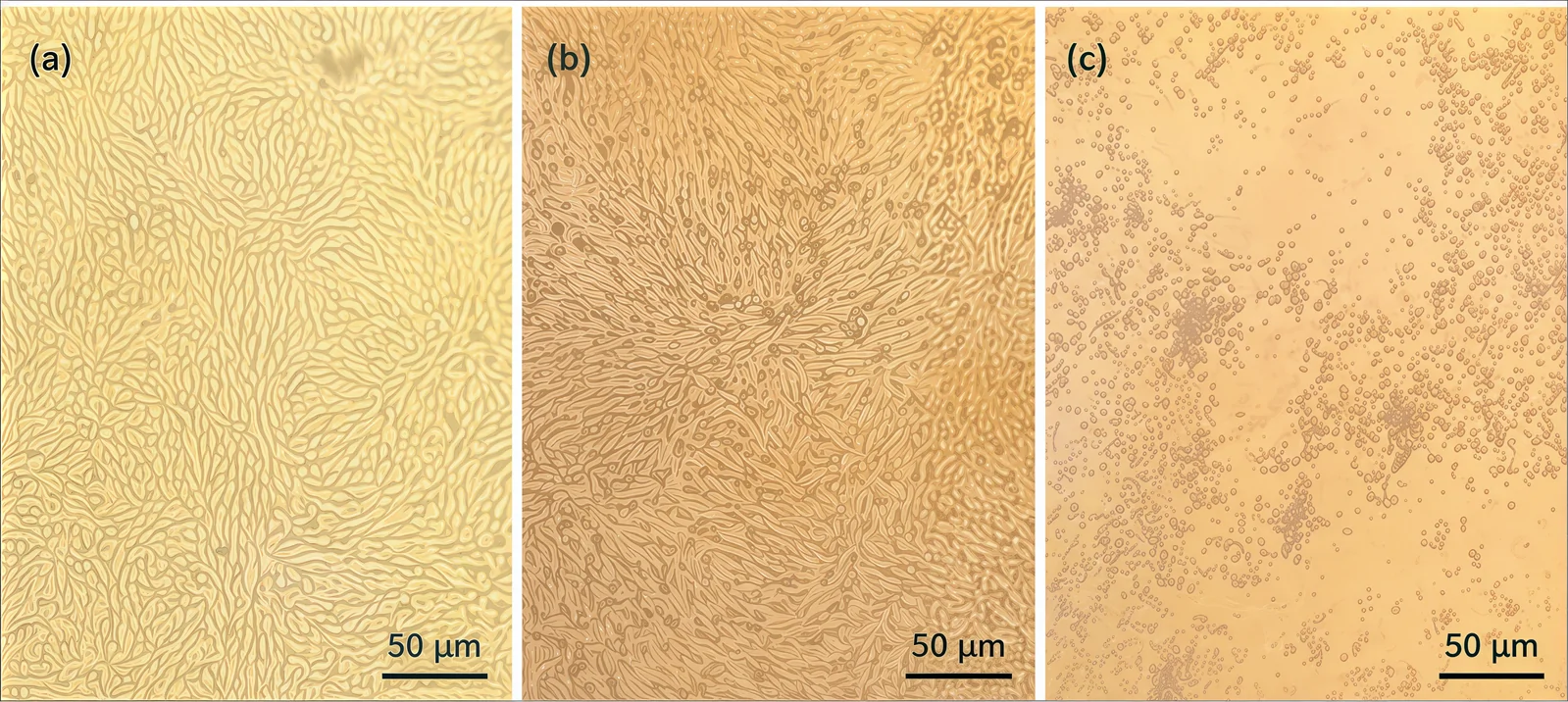
Stages of LSDV isolation in Vero cells illustrate morphological changes: (a) uninoculated Vero cell, (b) CPE at day 4, and (c) CPE at day 7.

**Figure 4:**
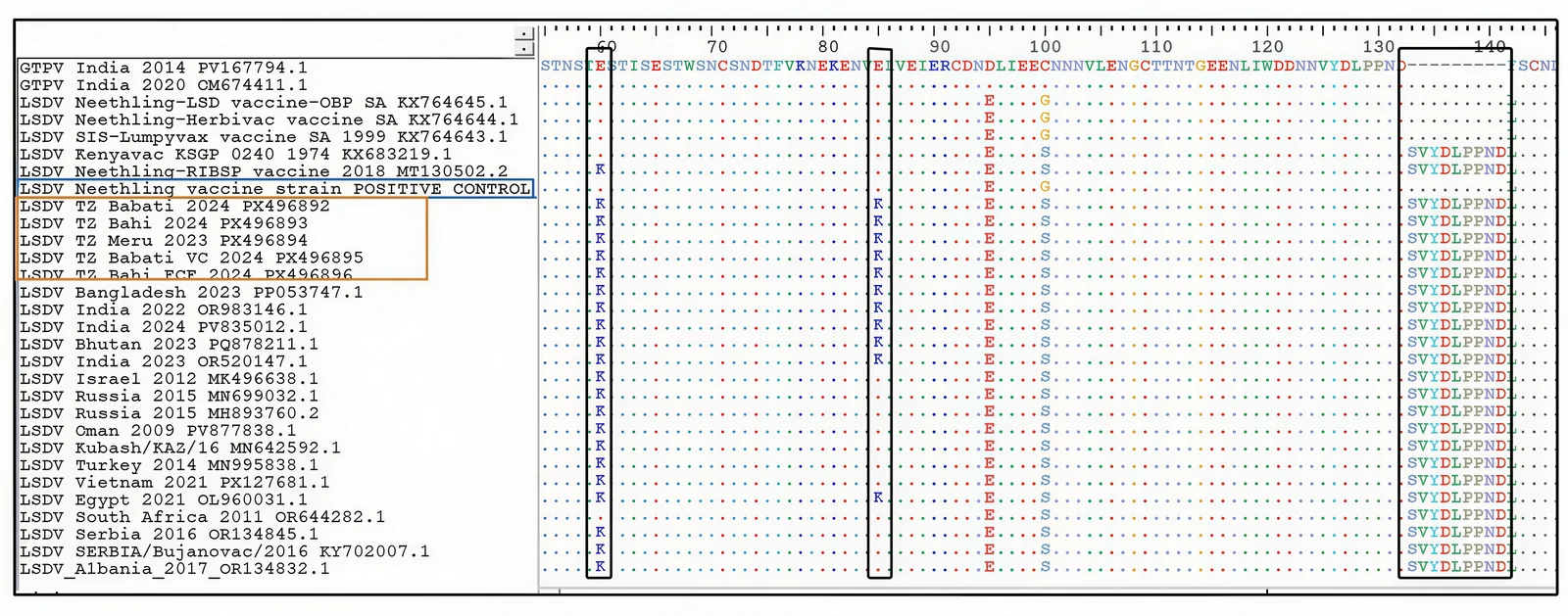
Multiple sequence alignments of the deduced amino acid sequences of the *EEV126* glycoprotein gene of LSDV. Note: LSDV strains from Tanzania were highlighted in a red rectangular box, whereas the vaccine commercially available was highlighted in a blue rectangular box. The regions are different from the vaccine strain that was highlighted in the black box.

**Figure 5:**
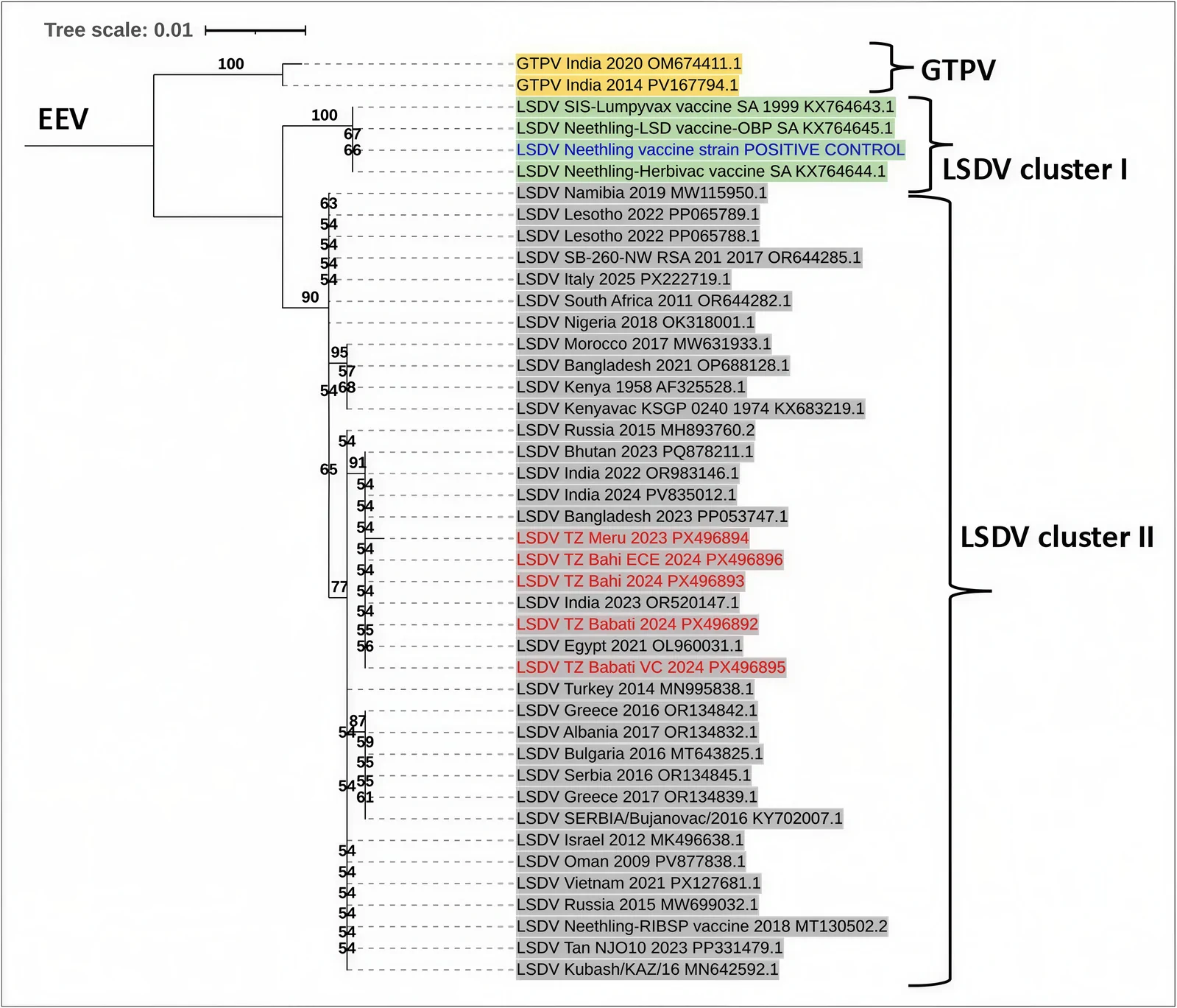
Phylogenetic analysis of the EEV126 glycoprotein gene based on nucleotide sequences demonstrating the relationships of Tanzanian LSDV strains, the available vaccine strain (positive control), and reference LSDV strains (vaccine and other strains) retrieved from the GenBank database. Note: Tanzanian field isolates obtained in this study were highlighted in red, and the positive control was highlighted in blue. GTPV sequences were used as outgroup sequences. The tree was constructed by the maximum likelihood method, utilizing the Tamura 3-parameter model in MEGA software.

**Figure 6:**
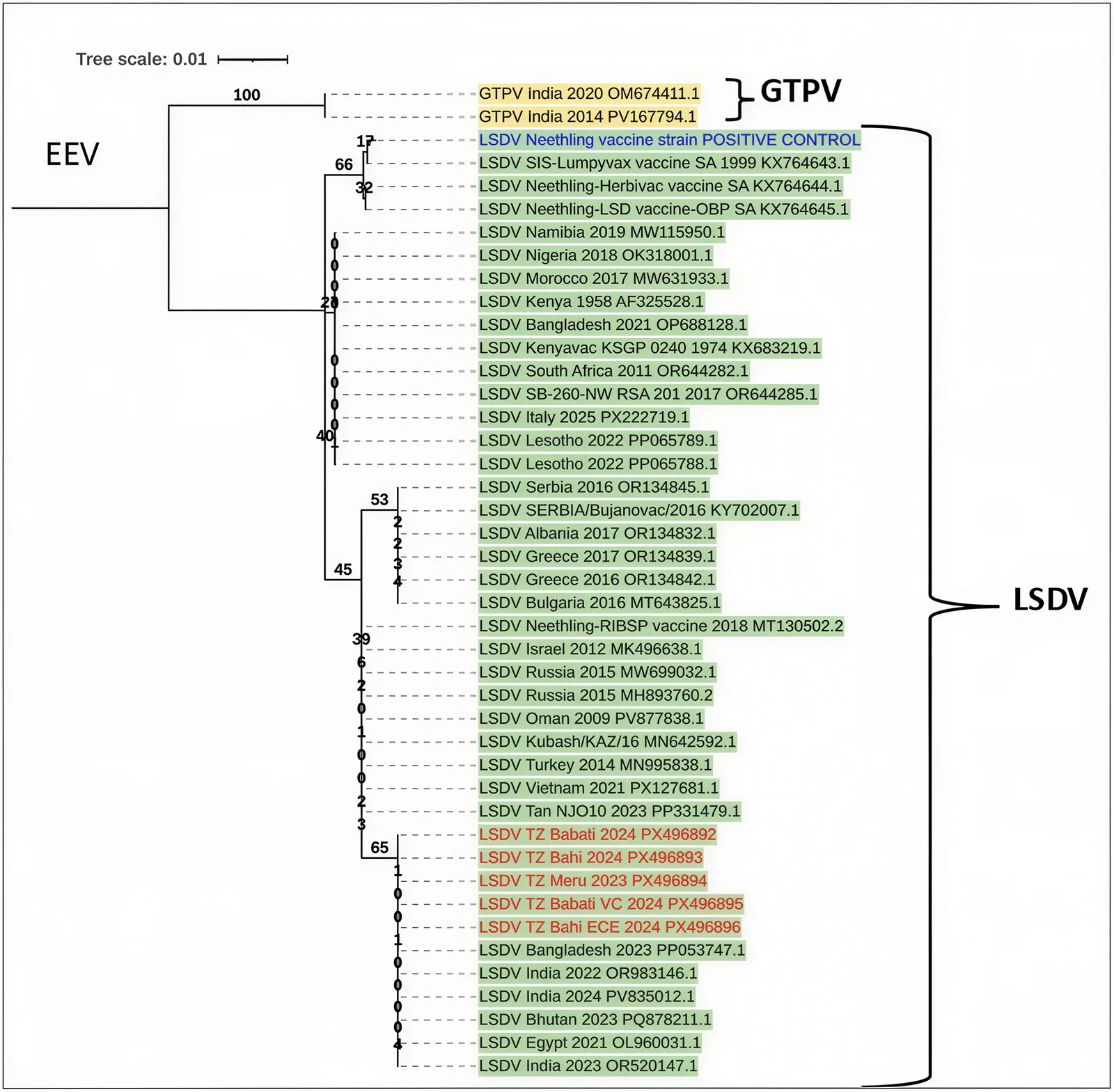
Phylogenetic analysis of EEV126 glycoprotein amino acid sequences illustrating the relationships of Tanzanian LSDV strains (highlighted in red), the available vaccine strain (positive control was highlighted in blue), reference LSDV strains, and the Neethling vaccine reference strains retrieved from the GenBank database.

**Figure 7:**
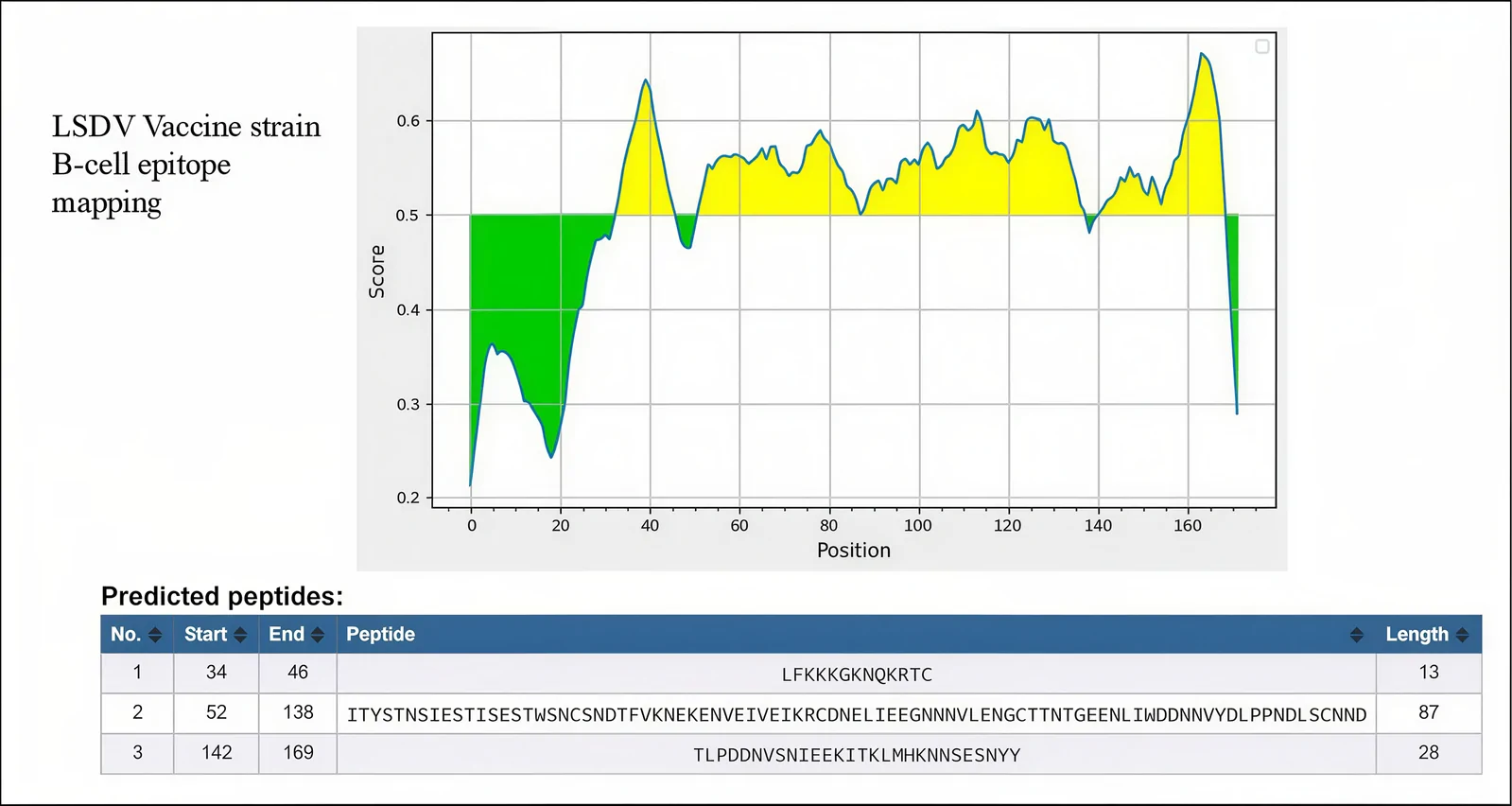
Predicted B-cell epitopes in the EEV126 protein of the LSDV vaccine strain.

**Figure 8:**
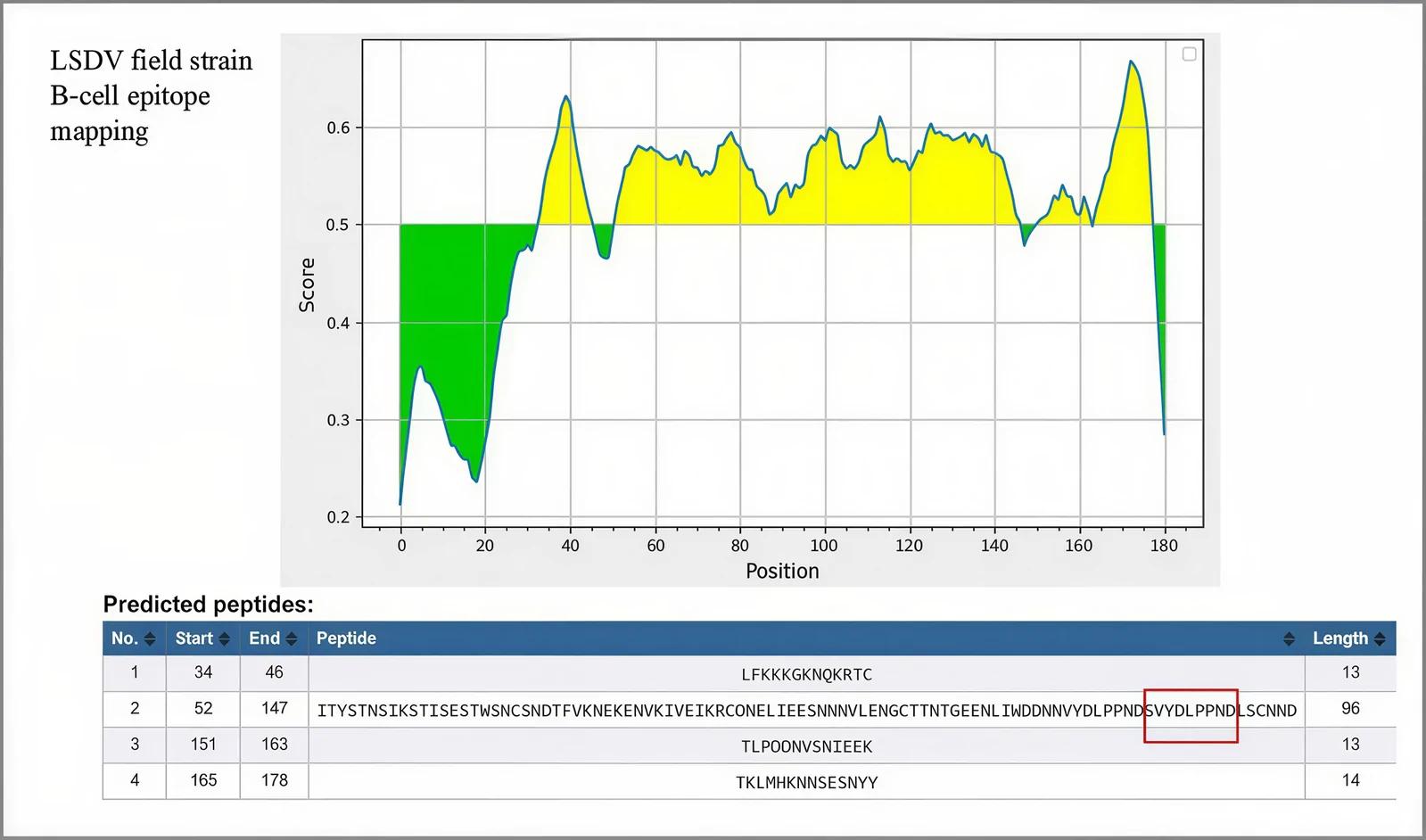
Predicted B-cell epitopes in the EEV126 protein of the LSDV Tanzanian field strain.

**Figure 9:**
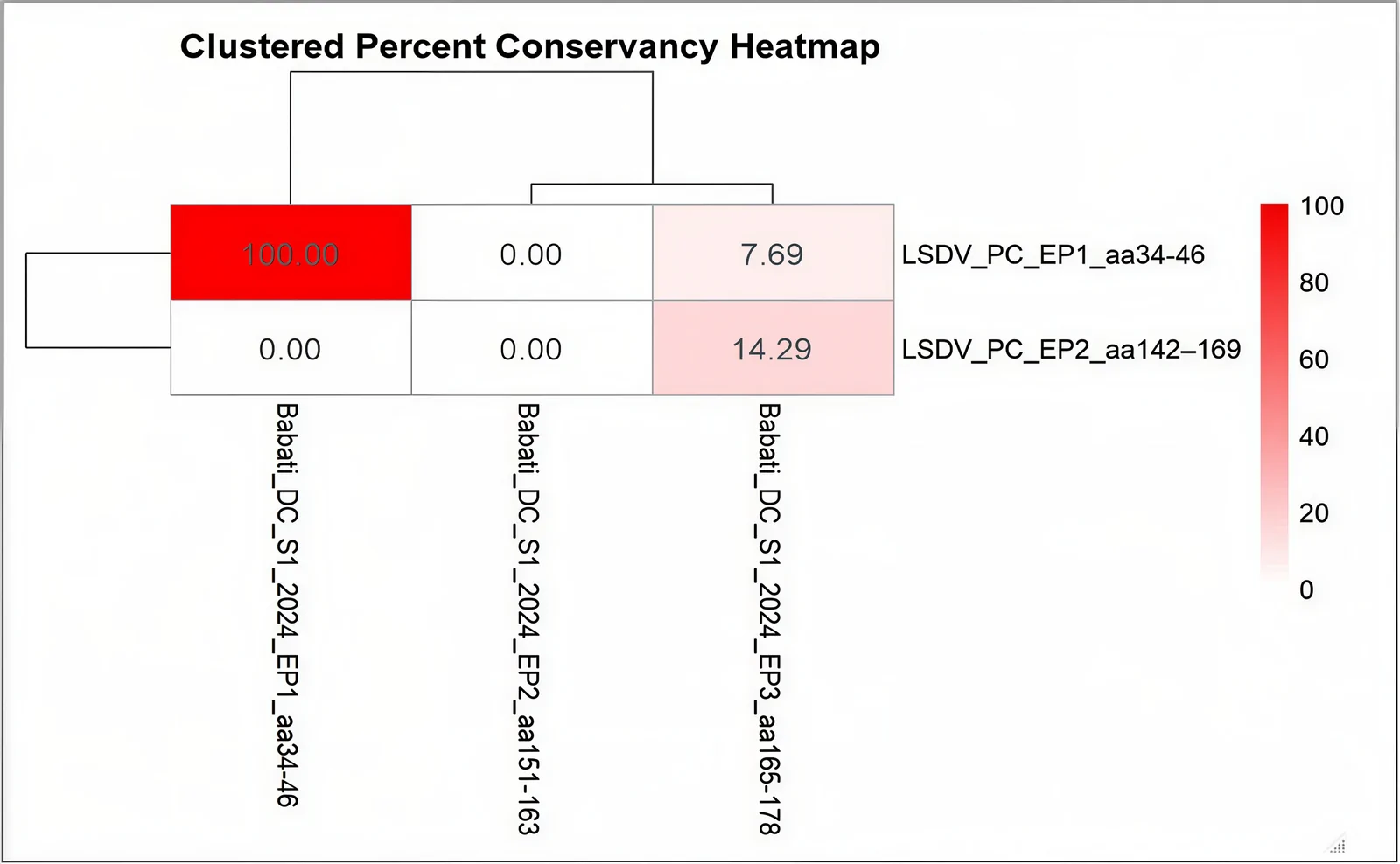
Sequence conservation of predicted B-cell epitopes in the LSDV vaccine and the field strains.

**Figure 10:**
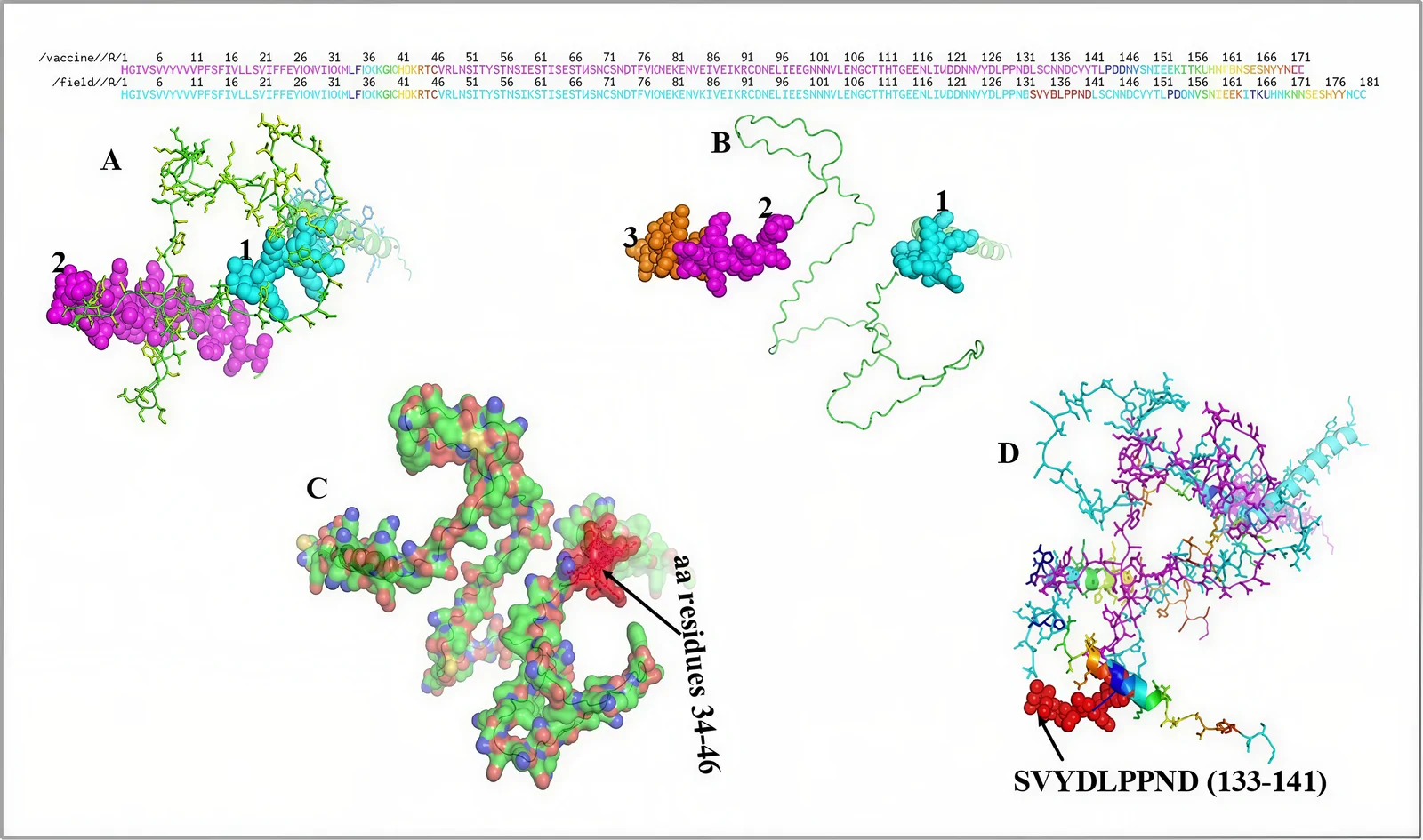
Surface mapping of predicted B-cell epitopes on the 3D structure of the EEV126 protein of LSDV. Numbers 1–3 indicate predicted B-cell epitope sites.Note: A: Predicted B-cell epitopes mapped onto the 3D structure of the EEV126 protein of the LSDV vaccine strain.B: Predicted B-cell epitopes mapped onto the 3D structure of the EEV126 of the LSDV Tanzanian field strainC: Mapping of the epitope showing 100% sequence conservation onto the overlay structure of the EEV126 protein from the LSDV vaccine and Tanzanian field strains D: Structural mapping of the 9-amino acid insertion (SVYDLPPND) onto the overlay 3D structure of the EEV126 protein of the LSDV field strain.

**Table 1:** Embryo survival across passages of virus in embryonated chicken eggs.

Passage	Number of eggs inoculated	Days post-inoculation (PI)	Embryo survival	Notes
First inoculation	15	Day 1–5	All embryos alive	No deaths observed
First passage	15	Day 1–5	All embryos alive	No deaths observed
Second passage	15	Day 1–3	All embryos alive	No deaths observed
		Day 4	2 embryos from one sample died	CAM from these eggs were harvested
		Day 5	13 embryos alive	All remained embryos were alive

**Table 2:** Percentage amino acid sequence identity between Tanzanian isolates and commercial vaccine strains used for cattle vaccination in Tanzania.

LSDV isolates	LSDV commercially available vaccine	LSDV Bahi PX496893	LSDV Babati PX496892	LSDV Meru PX496894	LSDV Babati VC PX496895	LSDV Bahi ECE PX496896
LSDV commercially available vaccine		92.21	92.21	92.21	92.21	92.21
LSDV Bahi PX496893	92.21		100	100	100	100
LSDV Babati PX496892	92.21	100		100	100	100
LSDV Meru PX496894	92.21	100	100		100	100
LSDV Babati VC PX496895	92.21	100	100	100		100
LSDV Bahi ECE PX496896	92.21	100	100	100	100	

VC = Vero cell, ECE = Embryonated chicken eggs.

## Data Availability

The data used during the current study will be made available on request.

## List of abbreviations

CAM, chorioallantoic membrane; CPE, cytopathic effect; ECE, embryonated chicken eggs; EEV, extracellular enveloped virus; FBS, fetal bovine serum; gm, grams; h, hours; HEET, Higher Education for Economic Transformation; IEDB, immune epitope database; JTT, Jones–Taylor–Thornton; LSD, lumpy skin disease; LSDV, lumpy skin disease virus; MEGA, Molecular Evolutionary Genetics Analysis; MEM, minimum essential media; min, minutes; ml, milliliter; µl, microliter; PDB, protein data bank; pLDDT, predicted local distance difference test; sec, seconds.
